# Identification of sex chromosomes in *Eremiasvelox* (Lacertidae, Reptilia) using lampbrush chromosome analysis

**DOI:** 10.3897/CompCytogen.v13i2.34116

**Published:** 2019-05-14

**Authors:** Artem P. Lisachov, Svetlana A. Galkina, Alsu F. Saifitdinova, Svetlana A. Romanenko, Daria A. Andreyushkova, Vladimir A. Trifonov, Pavel M. Borodin

**Affiliations:** 1 Institute of Cytology and Genetics SB RAS, Acad. Lavrentiev Ave. 10, Novosibirsk 630090, Russia; 2 Saint Petersburg State University, Universitetskaya Emb. 7–9, Saint Petersburg 199034, Russia; 3 Herzen State Pedagogical University of Russia, Moika emb. 48, Saint Petersburg 191186, Russia; 4 Institute of Molecular and Cellular Biology SB RAS, Acad. Lavrentiev Ave. 8/2, Novosibirsk 630090, Russia; 5 Novosibirsk State University, Pirogova Str. 2, Novosibirsk 630090, Russia

**Keywords:** meiosis, microdissection, sex chromosomes, lampbrush chromosomes, heterochromatin, lizard

## Abstract

Reptiles are good objects for studying the evolution of sex determination, since they have different sex determination systems in different lineages. Lacertid lizards have been long-known for possessing ZZ/ZW type sex chromosomes. However, due to morphological uniformity of lacertid chromosomes, the Z chromosome has been only putatively cytologically identified. We used lampbrush chromosome (LBC) analysis and FISH with a W-specific probe in *Eremiasvelox* (Pallas, 1771) to unequivocally identify the ZW bivalent and investigate its meiotic behavior. The heterochromatic W chromosome is decondensed at the lampbrush stage, indicating active transcription, contrast with the highly condensed condition of the lampbrush W chromosomes in birds. We identified the Z chromosome by its chiasmatic association with the W chromosome as chromosome XIII of the 19 chromosomes in the LBC karyotype. Our findings agree with previous genetic and genomic studies, which suggested that the lacertid Z chromosome should be one of the smaller macrochromosomes.

## Introduction

Reptiles represent a good model system for studying the evolution of sex determination, since different reptiles possess different sex determination systems. In some groups of reptiles (e.g., crocodiles, some turtles, some geckos), the sex of the offspring is determined by the temperature of egg incubation (TSD, temperature sex determination) (Viets et al. 1993). In other groups, various genetic sex determination (GSD) systems are found, ranging from GSD without heteromorphic sex chromosomes to prominently heteromorphic sex chromosome systems, some of which originated independently in different lineages from different ancestral autosomal pairs (Pokorná and Kratochvíl 2016). In some cases, different sex determination systems occur even in closely related species (Koubová et al. 2014, Gamble et al. 2015).

Several reptile lineages have sex chromosome systems common to the whole family or infraorder. These lineages include iguanas (Pleurodonta, or Iguanidae sensu lato) (Rovatsos et al. 2014), advanced snakes (Caenophidia) (Rovatsos et al. 2015), monitor lizards (Varanidae) and probably the whole anguimorph lizard group (Rovatsos et al. 2019), and lacertid lizards (Lacertidae) (Rovatsos et al. 2016a, b; see also Srikulnath et al. 2014). Comparative and evolutionary cytogenetics and genomics can determine the identities of different reptile sex chromosomes, and their homologs or syntenic chromosome regions in other animals’ genomes (Deakin and Ezaz 2019).

Lacertids have a ZZ/ZW (female heterogametic) sex chromosome system. Their sex chromosomes were discovered in the early 1970s and have since been extensively studied (Ivanov and Fedorova 1973, Olmo et al. 1986, 1987, Odierna et al. 1993, Pokorná et al. 2011, Giovannotti et al. 2018). The W chromosome of lacertids is highly degenerate, and therefore can be easily identified in the karyotypes of most species by its size and/or differential staining and/or repetitive DNA content (Capriglione et al. 1994, Pokorná et al. 2011, Matsubara et al. 2015), although its exact size and DNA content vary strongly across species.

The lacertid Z chromosome is more difficult to identify. Lacertids typically have 18 pairs of extremely acrocentric or subtelocentric macrochromosomes, gradually decreasing in length, and a pair of microchromosomes (2n=38). The macrochromosomes can be roughly divided into two size groups: larger chromosomes 1–10 and smaller chromosomes 11–18 (Srikulnath et al. 2014). Differential staining techniques like G-banding, which gives chromosome-specific banding patterns in mammals, generally work poorly on reptiles.

Early studies yielded contradictory identifications of the lacertid “Z chromosome”: it appeared as one of the largest chromosomes in some ideograms, and as one of the small chromosomes in others (Olmo et al. 1986, Odierna et al. 1993). Srikulnath et al. (2014) identified a putative Z chromosome of *Lacertaagilis* Linnaeus, 1758 as chromosome 5, based on Hoechst staining patterns. Recent works by Giovannotti et al. (2017) and Schmid et al. (2019) showed putative Z chromosomes of *Acanthodactyluserythrurus* (Schinz, 1933) and *Lacertatrilineata* Bedriaga, 1886, identified by FISH with a telomeric probe and immunofluorescent localization of 5-methylcytosine, respectively, as small chromosomes.

Z-linked genes of many lacertid species were identified using transcriptome analysis and qPCR to detect genome regions with low coverage specific to one sex (Rovatsos et al. 2016a). Orthologues of all genes identified in various species are located in two microchromosomes in *Anoliscarolinensis* Voigt, 1832 (Kichigin et al. 2016). Rovatsos et al. therefore suggested that the lacertids share the same Z chromosome, which is probably small. However, they did not visualize it directly. Therefore, the Z chromosomes of lacertids have not yet been unequivocally identified cytologically.

In our study, we rely on the existence of a chiasmatic association between the Z chromosome and the easily detectable W chromosome in meiotic prophase I. To visualize the sex bivalent, we obtained lampbrush chromosome (LBC) preparations from the rapid racerunner (*Eremiasvelox* (Pallas, 1771)). LBCs represent a specific condition of meiotic chromosomes which is found in maturing oocytes of birds, reptiles, fishes, and amphibians (Callan 1986). They are widely used in amphibian and bird cytogenetics. LBC spreads from lacertids have been reported before (Lukina 1994), but the sex chromosomes were not identified. The W chromosome of *E.velox* was previously studied by Pokorná et al. (2011). It is relatively large, but totally heterochromatic and harbours many satellite repeat sequences. To confirm the identification of the sex bivalent, we performed FISH with a microdissected probe of the W chromosome, obtained from the mitotic metaphase plate.

## Material and methods

### Samples and DNA barcoding

Two adult and two juvenile *E.velox* were obtained from private keepers. The adults were used for LBC preparation, and the juveniles were used for fibroblast cultures. All manipulations with live animals and euthanasia were approved by the Saint Petersburg State University Ethics Committee (statement #131-03-2) and the Institute of Molecular and Cellular Biology Ethics Committee (statement #01/18 from 05.03.2018). To confirm the species identity, we carried out DNA barcoding. DNA was extracted from ethanol-preserved blood of one of the adult specimens by the conventional phenol-chloroform technique (Sambrook et al. 1989). Primers and PCR conditions for the amplification of the fragment of the mitochondrial COI gene were as described earlier (Nagy et al. 2012). After PCR, the products were purified by electrophoresis in 1% agarose gel, cut from the gel and extracted by a commercial DNA gel extraction kit (BioSilica, Novosibirsk, Russia). The amplicons were Sanger sequenced using the BigDye3.1 reagent (ThermoFisher Scientific, USA), and the sequence was processed using MEGA7 (https://megasoftware.net). Then the sequence was analyzed using the distance-based and tree-based identification tools of the BOLD v.4 database (Ratnasingham and Hebert 2007; http://boldsystems.org/).

### Lampbrush chromosome preparation

LBCs of *E.velox* were manually dissected from previtellogenic and early vitellogenic oocytes (each ovary contained 15–16 such oocytes) using the standard avian lampbrush technique described by Saifitdinova et al. (2017) with slight modifications: namely, MgCl_2_ was excluded from the buffer solutions and EDTA was added to a final concentration of 0.01% to better disrupt the oocyte nucleus content. After centrifugation, preparations were fixed in 2% paraformaldehyde, then in 50% and in 70% ethanol. After dehydration in 96% ethanol, preparations were air-dried and mounted in antifade medium (1–1.2% DABCO, 2× SSC, 50% glycerol) with DAPI (50 ng/mL). After acquiring the DAPI and phase contrast images, the preparations were washed in 2× SSC, dehydrated in ethanol series (70%, 80%, 96%), air-dried and subjected to FISH.

### Cell cultures and metaphase chromosome preparation

Primary fibroblast cell lines were established in the Laboratory of Animal Cytogenetics, the Institute of Molecular and Cellular Biology, Russia, using enzymatic treatment of tissues as described previously (Stanyon and Galleni 1991, Romanenko et al. 2015). All cell lines were deposited in the IMCB SB RAS cell bank (“The general collection of cell cultures”, 0310-2016-0002). Metaphase chromosome spreads were prepared from chromosome suspensions obtained from early passages of primary fibroblast cultures as described previously (Yang et al. 1999, Graphodatsky et al. 2000, 2001).

### Microdissection and FISH

Candidate chromosomes were manually microdissected from the Giemsa-stained metaphase plates using an Olympus IX-51 microscope equipped with an Eppendorf Transferman NK2 micromanipulator. Since the W chromosome does not have specific morphological features, we dissected 26 chromosomes of appropriate size from 3 metaphase plates. The dissected chromosomes were amplified and labelled with biotin- and digoxigenin-dUTP (Roche) using the commercial GenomePlex Whole Genome Amplification (WGA-1) kit (Sigma). The probes obtained were checked and characterized by FISH on metaphase chromosome preparations as described in Liehr et al. (2017). The recognized W chromosome-specific probe was used for FISH on LBCs, which was carried out as described above, omitting the RNAse and pepsin treatment stages.

### Microscopy and image processing

DAPI and phase contrast images were acquired with a Leica DM4000B microscope installed at the “Chromas” Resource Centre, Saint Petersburg. The FISH preparations were analyzed with an Axioplan 2 Imaging microscope (Carl Zeiss) equipped with a CCD camera (CV M300, JAI), CHROMA filter sets, and the ISIS4 image processing package (MetaSystems GmbH). The brightness and contrast of all images were enhanced using Corel PaintShop Photo Pro X6 (Corel Corp). The lengths of the LBCs were measured using MicroMeasure 3.3 software (Reeves 2001).

## Results

**Figure 1.**
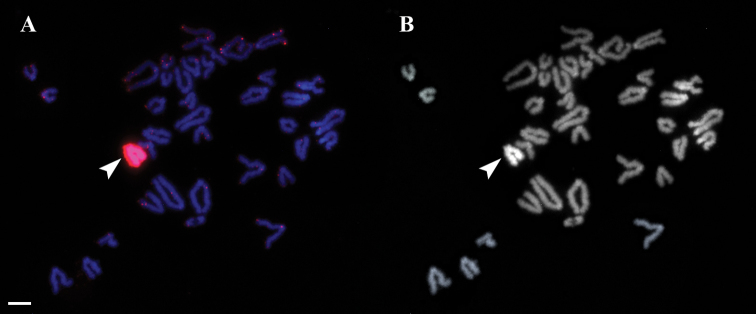
FISH with the microdissected W-specific probe on mitotic chromosomes of *Eremiasvelox***A** DAPI (blue), W-specific probe (red) **B** DAPI channel separately. Arrowhead indicates W chromosome. Scale bar: 10 μm.

The DNA sequence (GenBank accession number MK558359) showed that the specimens analyzed belong to the “eastern” clade of *E.velox* (the nominative subspecies *E.veloxvelox* (Pallas, 1771)). The mitotic karyotype of the lizards studied was typical of Lacertidae and was in agreement with previous studies (Kupriyanova and Arronet 1969; Pokorná et al. 2011). It consisted of 38 uniarmed chromosomes gradually decreasing in length. The W chromosome was DAPI-positive, and one of the microdissected probes showed a very strong hybridization signal on it (Fig. 1). It also gave several additional hybridization signals in the telomeres and centromeres of some autosomes, but no other chromosome showed a hybridization signal across its whole length. This probe was concluded to be W-specific.

The contents of the oocyte nuclei after the removal of the nuclear envelope formed a dense ball, and its full dispersal was more difficult to achieve than with birds and amphibians. Thus, most LBC sets showed insufficient spreading, and only one finely spread and complete chromosome set was obtained. The lampbrush karyotype of *E.velox* consisted of 19 bivalents, with the bivalent XIX (the only microchromosome) significantly smaller than the others (Suppl. material 1: Fig. S1). This agrees with the mitotic karyotype. The bivalents typically had one or two terminal or subterminal chiasmata. The total number of chiasmata per spread was estimated as 35 to 38. Interestingly, the microchromosomal bivalent (XIX) had two chiasmata.

**Figure 2.**
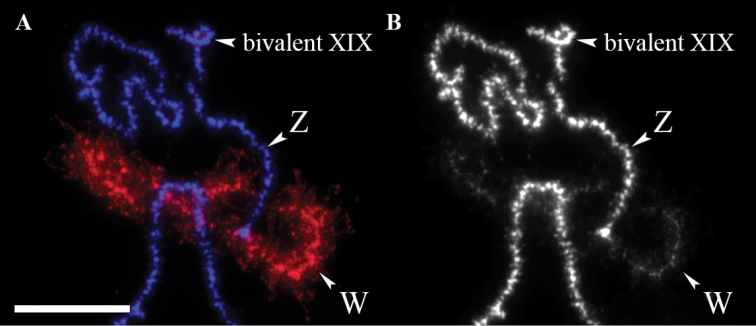
FISH with the microdissected W-specific probe on lampbrush sex bivalent of *Eremiasvelox*. **A** DAPI (blue), W-specific probe (red) **B** DAPI channel separately. Scale bar: 15 μm.

**Figure 3.**
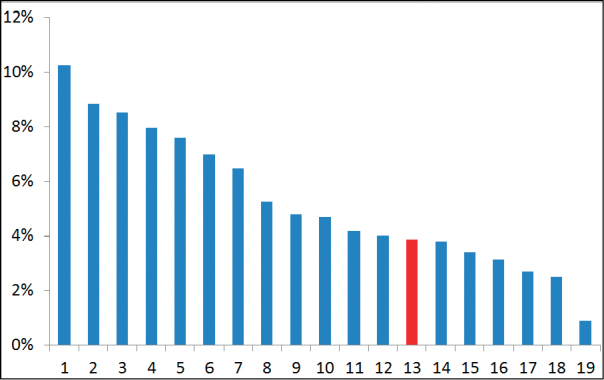
Ideogram of lampbrush karyotype of *Eremiasvelox*. Red indicates Z chromosome. X axis indicates size ranks. Y axis indicates relative length.

Although LBCs were isolated from previtellogenic oocytes, prominent lateral loops were absent on most bivalents, which is in accordance with a previous observation made in lacertids by Lukina (1994). This fact probably reflects that the oocytes which are large enough for LBC preparations are at relatively late diplotene stages in small lizards (Lukina 1994). In one of the bivalents, the homologues were different in length and chromatin state. One of the homologues consisted of dense chromomeres, resembling other chromosomes. The other homologue was decondensed and showed long chromatin loops. The W-specific probe labelled the decondensed homologue, thus confirming that this is the sex bivalent (Fig. 2). The sex bivalent had only a single chiasma, which was located terminally, suggesting a physically very short pseudoautosomal region. The measurements of the relative lengths of the LBCs showed that the Z chromosome is chromosome XIII in the lampbrush karyotype, thus belonging to the fraction of small chromosomes (Fig. 3).

## Discussion

For many years, amphibian and avian LBCs have been serving as a spectacular model for studying chromosome organization and genome functioning. In squamate reptiles, which also have a hypertranscriptional type of oogenesis, LBCs have scarcely been studied before. The initial descriptions of LBCs of *Lacertaagilis*, *Zootocavivipara* (Lichtenstein, 1823), *Darevskiaarmeniaca* (Méhely, 1909) and *Podarcistauricus* (Pallas, 1814) were made by Lukina (1994). However, no full karyotypes were described and the sex chromosomes were not identified. We are the first to describe the complete lacertid karyotype in the lampbrush form, and identify the sex bivalent by a molecular cytogenetic approach.

We noted above that most lampbrush bivalents had one or two chiasmata. The observed number exceeds the mean numbers of recombination nodules in male meiosis in *Darevskia* Arribas, 1999, identified by immunolocalization of SYCP3 and MLH1 proteins at pachytene, which equaled 24–29 in different species (Spangenberg et al. 2017, 2019). In particular, the occurrence of two chiasmata, like those observed in bivalent XIX (Fig. 2), is extremely rare in the microchromosomes of male lizards (Lisachov et al. 2017, 2019). This suggests the occurrence of more crossovers in female than male meiosis in lacertids (heterochiasmy). Different types of heterochiasmy, including more crossovers in one sex than in another, and/or different crossover localizations, are known in many species (Mank 2009). However, since our sample size is limited to one spread, more data are required to draw firm conclusions about crossover numbers. The terminal and sub-terminal localization of most chiasmata is also consistent with the previously obtained data on lacertid lizards and many other animal species (Mézard et al. 2015).

The decondensed state of the heterochromatic W chromosome in *E.velox* contrasts with the lampbrush sex bivalents of birds, in which the heterochromatic W chromosome is much more condensed than the Z and autosomes (Solovei et al. 1993). Numerous lateral loops indicate that the W chromosome of *E.velox* is transcriptionally active at the lampbrush stage. Due to the transcriptional activity of LBCs, an enormous amount of RNA is synthesized in the oocyte nucleus, mainly of sequences that do not encode proteins, e.g. transposable and some satellite repeated sequences (Gaginskaya et al. 2009). These transcripts could have functions in regulatory mechanisms involved in embryonic development, epigenetic processes, maintaining chromatin structure, or other functions (Gaginskaya et al. 2009). More detailed analysis of sex chromosome behavior in meiosis in *E.velox* and other lacertids is required to determine whether the high transcriptional activity of the W chromosome is common to all lacertids, what these transcripts represent and their biological roles, what is the extent of “degeneration” and heterochromatinization of the W chromosome, and its possible “junk” repetitive sequences accumulated.

This study is the first unequivocal cytological identification of a lacertid lizard Z chromosome. The size ranks of LBCs do not always correlate precisely with the sizes of the mitotic chromosomes, or their relative genomic lengths (Daks et al. 2010). Given the similar sizes of the small macrochromosomes in the lacertid karyotypes (Srikulnath et al. 2014), the Z chromosome of the rapid racerunner may not be its 13^th^ largest chromosome, but it is evident that it belongs to the group of small macrochromosomes.

Our identification is in good agreement with the previous recent putative cytological identifications of Z chromosomes in *A.erythrurus* and *L.trilineata* (Giovannotti et al. 2017, Schmid et al. 2019) using FISH and immunostaining, and with the genetic identifications: in several lacertid species using the qPCR approach mentioned above (Rovatsos et al. 2016a), and in *Podarcismuralis* (Laurenti, 1768) using coverage differences between genome sequences from male and female samples (Andrade et al. 2019). Chromosome 5, which belongs to the group of large macrochromosomes and was suggested to be the sex chromosome in *L.agilis* (Srikulnath et al. 2014), is apparently not a sex chromosome in *E.velox*.

Identification of the *E.velox* sex chromosomes should lead to further studies of sex chromosome evolution and function in Lacertidae, including estimates of the extent of W chromosome genetic degeneration and its time course. Reliable identification of the *E.velox* Z chromosome will facilitate obtaining Z-derived chromosome-specific and region-specific probes for cytogenetic and genomic studies, including via LBC microdissection.

## Supplementary materials

Supplementary material 1 Figure S1 Copyright notice: This dataset is made available under the Open Database License (http://opendatacommons.org/licenses/odbl/1.0/). The Open Database License (ODbL) is a license agreement intended to allow users to freely share, modify, and use this Dataset while maintaining this same freedom for others, provided that the original source and author(s) are credited.Artem P. Lisachov, Svetlana A. Galkina, Alsu F. Saifitdinova, Svetlana A. Romanenko, Daria A. Andreyushkova, Vladimir A. Trifonov, Pavel M. Borodin
