# Supplementary figures and images for: Identification of sex chromosomes in Eremiasvelox (Lacertidae, Reptilia) using lampbrush chromosome analysis
Source: Comp Cytogenet. 2019 May 14;13(2):121–32. doi: 10.3897/CompCytogen.v13i2.34116 (PMC6529369; doi:10.3897/CompCytogen.v13i2.34116)

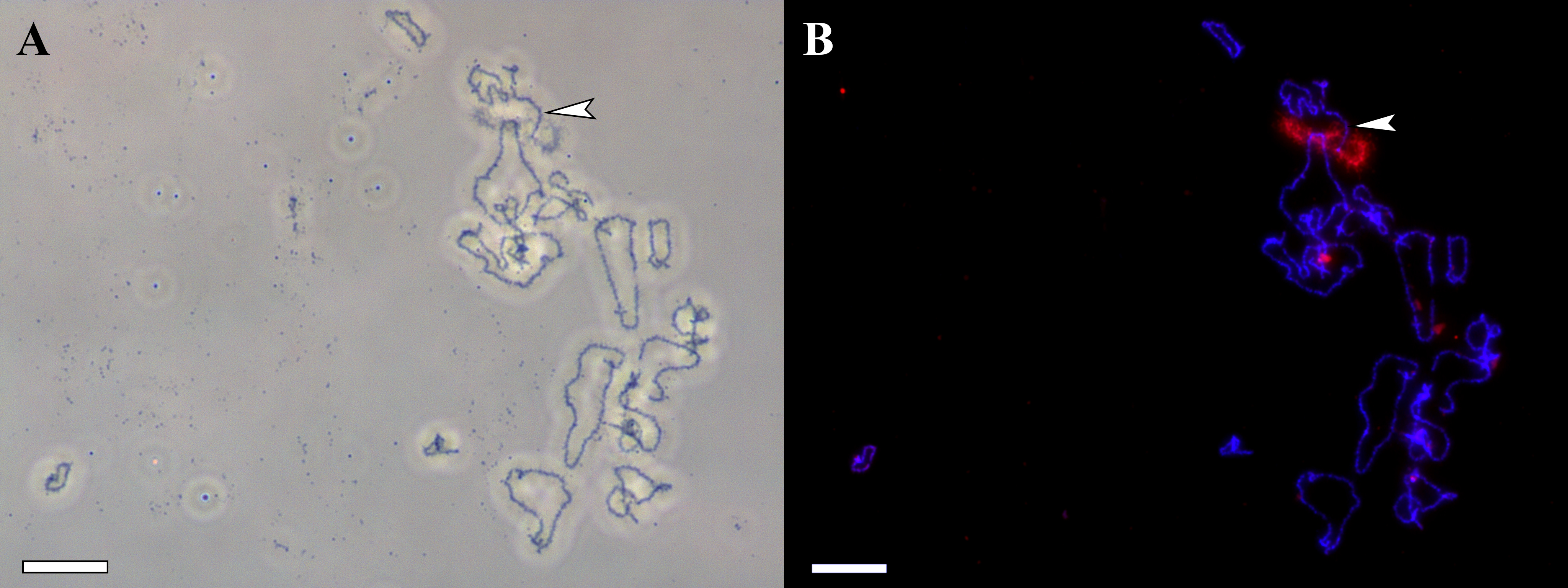

Supplement: Supplementary material 1 [file comparative_cytogenetics-13-121-s001.jpg]
